# Early Word Segmentation Behind the Mask

**DOI:** 10.3389/fpsyg.2022.879123

**Published:** 2022-05-09

**Authors:** Sónia Frota, Jovana Pejovic, Marisa Cruz, Cátia Severino, Marina Vigário

**Affiliations:** Center of Linguistics, University of Lisbon, Lisbon, Portugal

**Keywords:** early word segmentation, face mask, COVID-19, auditory speech, audiovisual speech, speech perception, prosodic edge

## Abstract

Infants have been shown to rely both on auditory and visual cues when processing speech. We investigated the impact of COVID-related changes, in particular of face masks, in early word segmentation abilities. Following up on our previous study demonstrating that, by 4 months, infants already segmented targets presented auditorily at utterance-edge position, and, using the same visual familiarization paradigm, 7–9-month-old infants performed an auditory and an audiovisual word segmentation experiment in two conditions: without and with an FFP2 face mask. Analysis of acoustic and visual cues showed changes in face-masked speech affecting the amount, weight, and location of cues. Utterance-edge position displayed more salient cues than utterance-medial position, but the cues were attenuated in face-masked speech. Results revealed no evidence for segmentation, not even at edge position, regardless of mask condition and auditory or visual speech presentation. However, in the audiovisual experiment, infants attended more to the screen during the test trials when familiarized with without mask speech. Also, the infants attended more to the mouth and less to the eyes in without mask than with mask. In addition, evidence for an advantage of the utterance-edge position in emerging segmentation abilities was found. Thus, audiovisual information provided some support to developing word segmentation. We compared 7–9-monthers segmentation ability observed in the Butler and Frota pre-COVID study with the current auditory without mask data. Mean looking time for edge was significantly higher than unfamiliar in the pre-COVID study only. Measures of cognitive and language development obtained with the CSBS scales showed that the infants of the current study scored significantly lower than the same-age infants from the CSBS (pre-COVID) normative data. Our results suggest an overall effect of the pandemic on early segmentation abilities and language development, calling for longitudinal studies to determine how development proceeds.

## Introduction

Language includes auditory and visual cues relevant to language learning. The COVID-19 pandemic has affected communication and interaction in multifarious ways, most prominently by introducing ubiquitous face mask use. Such changes affected the auditory and visual cues available to the young language learner. In the current study, we investigated the impact of COVID-19-related changes, particularly of face masks, on infants' ability to extract potential word forms from the speech stream. Specifically, we examined whether word segmentation abilities differ between auditory only and audiovisual speech delivered without and with a face mask. In addition, we also examined whether COVID-19 related changes, which prominently include continued exposure to altered speech cues, might have hindered the development of word segmentation.

In everyday communication, speech is perceived auditorily, i.e., through hearing, and visually, through the speaker's articulatory, facial, and body movements. A bulk of research has examined how infants process auditory speech (e.g., Kuhl, 2004, for a review). However, language and adult-infant interactions tend to occur in face-to-face communication. During the 1st year of life, infants are dominantly exposed to human faces in comparison to other stimuli in their environment (Fausey et al., 2016; Jayaraman and Smith, 2018), and research has demonstrated infants' early sensitivity to visual speech (e.g., Kuhl and Meltzoff, 1984; Patterson and Werker, 1999; Lewkowicz and Hansen-Tift, 2012; Tomalski et al., 2013; Morin-Lessard et al., 2019; Pejovic et al., 2019). Such early sensitivity to visual speech has been proved important in infants' language development, namely, in language discrimination abilities (Weikum et al., 2007; Sebastián-Gallés et al., 2012), learning of phonemic contrasts (Teinonen et al., 2008), processing of stress (Cruz et al., 2020), and processing of familiar words (Weatherhead and White, 2017). When processing faces, infants dominantly attend to the eyes and the mouth of a speaker (e.g., Hunnius and Geuze, 2004). Attention to the eyes at 6–12 months of age is related to infants' concurrent social and communication skills (Pons et al., 2019), while, in 2–3-year-old toddlers, it is related to larger vocabulary (Sekiyama et al., 2021). Interestingly, attention to the mouth at 6 months of age is related to larger expressive vocabulary (Tsang et al., 2018), and recent research has shown a trend toward increased attention to the mouth and larger expressive vocabulary in 9–14-month-old infants (Morin-Lessard et al., 2019). Overall, previous research indicates that speech perception is inherently multisensory, and infants integrate auditory and visual cues very early in development (Choi et al., 2018).

When the world encountered COVID-19 health protection measures, usage of a face mask in everyday communication in public/work/school areas was widely accepted. Apart from the obvious benefit in protecting the population from the virus, usage of the face mask has raised questions about its effects on face-to-face communication. The face mask visually covers the mouth and creates an obstacle in assessing visual speech. In addition to changes in the visual speech signal, the acoustic speech signal is also altered. Recent research has focused on understanding the potential face mask impact on auditory and visual speech processing. Some studies indicate that face masks degrade the acoustics of speech in different degrees, depending on the mask material (for a review, see Thibodeau et al., 2021). Such degradation can be mild ~2d B with a surgical mask and an FFP2 mask (Bottalico et al., 2020; Cruz et al., 2022) to more intense ~20 dB with transparent masks (Atcherson et al., 2020). Face masks, again depending on the material, affect other aspects of the acoustic signal, such as power distribution, spectral tilt, and timing, which are particularly affected by the FFP2 face mask (Rahne et al., 2021).

An increasing number of recent studies sought to answer how the face mask affects speech processing in adults, children, and infants. In adults, the degraded acoustic signal directly decreases speech recognition in noise, which can be improved if adults have access to visual cues *via* a transparent mask. Yet, the improvement is not at the level of speech delivered without any mask (Thibodeau et al., 2021). Adult studies also suggest that face masks do not alter all aspects of speech processing. For instance, the following aspects have been shown to be negatively impacted: accuracy of word/sentence translation (Rahne et al., 2021), accuracy of speech recognition (e.g., Magee et al., 2020; Yi et al., 2021), listening effort (Haider et al., 2022), especially for hearing-impaired populations (Lee et al., 2022), intelligibility in noisy environments (Brown et al., 2021), or cortical tracking/reconstruction of audiovisual speech, especially reconstruction of high-level segmental features (i.e., phoneme and the word onset) in more challenging listening conditions (Haider et al., 2022). Interestingly, if speech is not placed in a noisy/challenging environment, intelligibility is usually intact (Magee et al., 2020; Brown et al., 2021; Rahne et al., 2021). In addition, a face mask did not affect adults' performance in a gaze-cuing task (Dalmaso et al., 2021). Given the alterations in the visual and/or acoustic speech signal and their impacts on adult speech processing, researchers have also raised the question whether face masks have an impact on speech perception in infants (e.g., Carnevali et al., 2021; Lewkowicz, 2021; Yeung et al., 2021).

A handful of studies tackled the effect of face-masked speech on early speech processing. In Singh et al. (2021), 2-year-old children were tested in a familiar word recognition task in three conditions: seeing a speaker with no mask, with an opaque mask (i.e., a surgical face mask), and a transparent mask (i.e., a face shield). The study revealed that children can recognize words in the no-mask and opaque-mask condition, but not in the transparent-mask condition. Authors argued that speakers wearing an opaque mask might compensate occlusion of the mouth by employing additional visual cues, such as eye movements, hence providing more (visual) speech information. However, in the case of the transparent face shield, the plastic material might notably alter the acoustic cues to impair early word recognition and the reflection, and refraction caused by the plastic material also affects the visual percept, which is optically distorted. Another study examined whether a face mask modulates natural mother-infant interaction. It was observed that wearing a face mask did not affect face-to-face mother-infant interaction during a free-play session in 5–19-month-old infants (Tronick and Snidman, 2021). However, masking has been shown to affect recognition of unfamiliar voices and their mapping to faces: 12-month-old infants were not able to recognize an unfamiliar speaker's voices when faces were partially occluded, unlike 24-month-old infants (Orena et al., In Press). Moreover, face masks have been shown to degrade speech understanding in children, namely consonant recognition, similarly to adults (Lalonde et al., In Press).

Apart from studies that directly measured the effect of face masks on language processing, some studies indicated that language development during the pandemic might as well be affected. For instance, a UK study analyzed language learning in 8–36-month-old infants growing up during the pandemic. The authors compared those infants that continued attending nursery with those that stayed at home and concluded that infants from a lower socioeconomic background had their receptive vocabulary growth boosted if attended nursery (Davies et al., 2021). The study suggested that activities in the nursery are especially beneficial for those infants that are coming from a more challenging background. Another large-scale study examined effects on language development of at-home activities, specifically parental interaction, during the first lockdown in 2020. It revealed that infants' vocabulary development was increased if parents were reading more to infants, while reducing the infants' exposure to passive screen time. The authors also argued that the results could be explained by parents' increased sensitivity to infants' development due to spending more time with them (Kartushina et al., 2022). These studies suggest that language development can be directly or indirectly modulated by changes that occurred during the pandemic. Another study suggested that even overall cognitive development could have been impaired during the pandemic times. Specifically, the study compared children's cognitive development during 2020 and 2021 — the period when most COVID-19 measures were placed in the US— with data collected in between 2011 and 2019. Cognitive development was measured *via* the Mullen Scales of Early Learning. The authors observed that children born in 2020-2021 demonstrated reduced cognitive skills (e.g., verbal, motor, and overall cognitive performance) compared to children born pre-pandemic. This reduction was particularly present for males and children raised in families with lower socioeconomic status (Deoni et al., 2021).

To summarize, recent studies have suggested that changes in everyday activities during the pandemic might affect language development (Davies et al., 2021; Deoni et al., 2021). The use of face masks, in particular, and related changes in the visual/acoustic speech cues, might alter certain aspects of speech processing, such as voice recognition, word recognition, and consonant recognition (Singh et al., 2021; Lalonde et al., In Press; Orena et al., In Press). However, developmental research is still in need to understand whether and when these potential effects take place during the early development, specifically, whether pandemic-related changes, namely, usage of face masks, affect infants' speech processing during the 1st year of life. The current study aims to address this question by testing infants' word segmentation abilities in presence or not of the face mask and compare segmentation abilities in infants born during the pandemic with earlier segmentation data collected in 2016-2017 (Butler and Frota, 2018). Segmenting linguistic units from the continuous speech stream is a precursor to infants' ability to identify word-like forms in speech and to match them to their referents, i.e., word learning (Bergmann and Cristia, 2016). In addition, word segmentation has been shown to support the development of syntax (e.g., Singh et al., 2012) and predict concurrent and later language development (Singh et al., 2012; Frota et al., 2020). Thus, word segmentation is a crucial milestone for infants' language development, and any alteration might reflect on later development. Moreover, a few studies suggest that speech segmentation is supported by visual speech cues in adults and infants. In adults, increased attention to the eyes seems to lead to better segmentation ability (Lusk and Mitchel, 2016). In infants, auditory and visual facial information is integrated to segment speech into phrases (de la Cruz-Pavía et al., 2019). To the best of our knowledge, only one study, with limited sample size, examined the role of audiovisual information in infants' word segmentation abilities and observed that these abilities are increased in the presence of audiovisual cues (Tan and Burnham, 2019). Therefore, it is possible that segmentation is affected if infants' access to auditory and/or audiovisual speech cues is altered by a face mask.

The current study has three main goals: 1) examine the momentary impact of mask use in early word segmentation abilities by contrasting the effects of speech delivered without and with a face mask; 2) establish whether audiovisual cues might provide additional support to facilitate infants' word segmentation by comparing auditory only and audiovisual speech; and 3) explore whether COVID-19-related changes in communication and interaction, which include continued exposure to altered speech cues, might have impacted the development of word segmentation by comparing segmentation abilities in infants born during the pandemic with segmentation data obtained prior to COVID-19. We conducted two word-segmentation experiments in 7–9-month-old European Portuguese learning infants: 1) An *Auditory experiment* where we tested infants' segmentation abilities in two conditions, with face-masked speech and speech produced without a mask; and 2) an *Audiovisual experiment* where we tested infants' segmentation abilities in the same two conditions. We followed up on work by Butler and Frota (2018), who examined emerging word segmentation abilities in European Portuguese learning infants from 4 to 10 months of age in an auditory task using a visual familiarization paradigm. We focused on 7–9-month-old infants because Butler and Frota (2018) demonstrated that the segmentation ability for words placed at utterance-edge position is well developed at that age, whereas segmentation for words in utterance-medial position is still developing. Specifically, the authors found that infants already at 4 months of age demonstrate segmentation for words at edge position, i.e., attend more to words they previously heard at utterance edges than to unfamiliar words, whereas words previously heard at utterance-medial position were still not clearly differentiated from unfamiliar words by 10 months of age. The second reason for focusing on 7–9-month-olds was that infants at that age tend to increase their attention to the mouth (e.g., Hunnius and Geuze, 2004; Lewkowicz and Hansen-Tift, 2012; Cruz et al., 2020; Pejovic et al., 2021), the region that is occluded by a face mask. In line with earlier work, segmentation abilities will be signaled by a consistent difference between looking times to previously heard (familiar) target words and unfamiliar target words, independently of the direction of preference. If speech delivered with a face mask has a direct and momentary effect on early word segmentation abilities, we predict a difference between the without-mask and with-mask experimental conditions. If audiovisual cues provide additional support to word segmentation, an advantage is expected for infants in the *Audiovisual experiment* relative to infants in the *Auditory experiment*. Finally, a difference between the findings from the current study, namely, in the *Auditory* experiment, and the earlier segmentation data collected in 2016-2017 with the same procedure, in the same lab by the same team, would indicate that continued exposure to altered speech cues together with COVID-related changes in everyday activities might have impacted the development of word segmentation abilities.

Our study was conducted in accordance with the recommendations of the European Union Agency for Fundamental Rights and the Declaration of Helsinki. The experimental procedures and informed consent protocols were approved by the ethics committee of the School of Arts and Humanities, University of Lisbon. Written informed consent was obtained from caregivers, in the infant experiments, and from adult participants, in the adult experiments, prior to data collection.

## Auditory Experiment

The word segmentation experiment from Butler and Frota (2018) study was used. The original study aimed to investigate the effect of prosodic edges on early segmentation abilities by presenting monosyllabic target word forms in one of the two prosodic conditions: at the utterance edge and at the utterance-medial position. The current study added the mask condition, i.e., the contrast between with a mask and without a mask, to the original design.

### Materials and Methods

#### Participants

Thirty-seven 7-to-9-month-old infants took part in the *Auditory* experiment. Eighteen infants were placed in the with-mask condition (mean age, 8.4 months; range, 7 months, 17 days – 9 months, 22 days; 10 females). Nineteen infants were placed in the without-mask condition (mean age, 8.5 months, range, 7 months, 5 days – 9 months, 7 days; 8 females). The infants in the two groups did not differ in their age [*t* (1, 35) = 1.08, *p* = 0.16]. Eight more infants were tested, but their data were not included in the analysis due to: crying or being fussy (4), not reaching the accumulated looking time criterion in the familiarization phase (2), and experiment failure (2). For all the tested infants, the parents reported no health-related issues, and no familial risks for language impairment. All the infants were born full-term (> 37 gestational weeks), with an APGAR score at the 5th min of more than 7, and weight of more than 2,500 g. The infants were recruited in the larger area of Lisbon, and all were acquiring European Portuguese as the only language. Information on the infants' average exposure to face masks in interaction with adults was assessed using an in-lab questionnaire, where the parents marked how many hours their infants were exposed to face masks. The parents could choose between 0 −2-h, 3 −5-h, and > 5-h everyday exposure. Eleven infants in the without-mask condition were estimated as being exposed to a face mask 0–2 h per day, and eight > 5 h per day. Ten infants in the with-mask condition were estimated as being exposed to a face mask 0–2 h per day, two to 3– 5 h, and five > 5 h (information for one infant was not provided). In addition, we also assessed the infants' exposure to European Portuguese *via* a language exposure parental questionnaire, similar to the questionnaire previously used to assess bilingual infants (Molnar et al., 2014). This questionnaire served not only to make sure that only monolingual European Portuguese infants were included but also to assess with how many people per week an infant was interacting with, and for how many hours. This information is important to control for variability across infants in their social interactions during the COVID-19 pandemic. We counted for each infant with how many people an infant was interacting with regularly on a weekly basis. We observed that the infants in without-mask condition, on average, interacted with 7.7 people, while 5 out of 19 infants attended a daycare. In the with-mask condition, the infants interacted with 6.7 people, and 6 out of 18 attended a daycare. Groups do not differ regarding number of people they regularly interact with [*t* (1, 35) = 1.5, *p* = 0.13]. We have also assessed concurrent language skills by using the European Portuguese version of the MacArthur Bates Communicative Development Inventory (CDI) short form for infants, meant for ages between 8 and 18 months (Frota et al., 2016). Overall communicative development was assessed by the European Portuguese version of the Communication and Symbolic Behavior Scales (CSBS) infant-toddler checklist aimed for infants 6–24 months of age (Frota et al., 2014). We observed no differences in CDI measures (i.e., scores and percentiles for comprehension and production abilities; all *p*s > 0.2). Regarding the CSBS measures^1^, we observed a marginal difference on a percentile rank on the social scale [*t* (1, 33) = 2, *p* = 0.05], with a higher social percentile rank in the with-mask condition (M = 63.4) than in the without mask (M = 44.1). On other CSBS measures (i.e., percentile on the symbolic scale, speech scale, and total score), the infants did not differ across conditions (all *p*s > 0.3).

#### Experiment Design

We compared the infants' segmentation abilities for pseudowords placed at the end of an utterance (i.e., utterance-edge position), which is a prosodically prominent position, with pseudowords placed in the middle of an utterance (i.e., utterance-medial position), which is a prosodically less prominent position. The task consisted of familiarization and test phases. During the familiarization, the infants heard target pseudowords embedded within short passages. After the familiarization, the infants were tested with isolated pseudowords that were present in the familiarization passages (familiar) and that were not present in the familiarization passages (unfamiliar). The infants were familiarized with two types of passages: 1) target pseudowords in utterance-medial position and 2) target pseudowords in utterance-edge position. To examine the effect of the face mask on the infants' segmentation abilities, the edge and medial positions were presented in two conditions: 1) *with a mask*, where the auditory stimuli in the familiarization phase were recorded, while the speaker wore a face mask and 2) *without a mask*, where the auditory stimuli in the familiarization phase were recorded while the speaker wore no mask. The test phase remained the same as in the original study, i.e., the infants were presented with isolated word forms produced without a mask.

#### Stimuli

The same four monosyllabic pseudowords from Butler and Frota (2018) were used: FUL ['fu], QUEU ['kεw], PIS['pi∫], and SAU ['saw]. These target word forms were embedded in carrier sentences either in utterance-edge or utterance-medial position to produce the familiarization stimuli. The latter consisted of the same short passages as in the original study. There were two passages for each pseudoword: one for utterance-edge and one for utterance-medial position. Passages consisted of six short sentences (with 9 to 11 syllables in length).

To replicate the original recordings as close as possible, we recruited the same female native European Portuguese speaker as in the original study and recorded her producing the sentences with and without an FFP2 (white) face mask as if she were talking to an infant. The speaker first heard the original sentence from the Butler and Frota (2018) study, and then she was recorded producing the same sentence. After one passage was recorded without the mask, the same passage was recorded with the mask, ensuring that the speaker replicated the same head movements and speech manners across with- and without-the-mask recordings. As in the original study, the stimuli were recorded with the same Sony unidirectional microphone (sampling frequency, 22,050 Hz). The speaker held the microphone in a close distance from the mouth. Each passage was edited in Audacity with a 500-ms pause between each sentence. All the sound stimuli used, as well as lists of the passages, are available at https://labfon.letras.ulisboa.pt/babylab/early_word_segmentation_behind_the_mask/supporting_materials.html.

The acoustic measurements and analysis of the stimuli are given in Table 1. Importantly, we observed, as in the original study, that, for both without- and with-mask conditions, prosodic properties at the utterance edge differ from utterance-medial position, with the edge showing more salient prosodic cues. In particular, differences were found in pre-boundary lengthening at edge position, as well as a greater pitch range, manifested by a pitch fall. The pitch fall is due to the presence of a major prosodic boundary, which is signaled by the occurrence of a low edge tone (annotated as L% following labeling conventions within the intonational phonology framework; Frota et al., 2015). Regarding the acoustic differences between the with- and without-mask conditions, detailed analysis is given in Cruz et al.'s (2022). The main findings from this study indicate that mean intensity was significantly lower in the with-mask condition (67.7 dB) than in the without-mask condition (68.9 dB). Moreover, mean intensity was higher in the edge relative to medial position in the without-mask condition, but not in the with-mask condition. Similarly, mean pitch was higher in edge position relative to medial in the without-mask condition, but not in the with-mask condition. Overall, the acoustic analysis suggests attenuation for some of the prosodic features that make the edge position more salient than medial when a speaker wore a face mask. The next step was to confirm whether the observed acoustic differences between the mask conditions were perceived by adult listeners to further understand the auditory features of the stimuli used in our experiment and their potential impact on speech perception. The next section describes a perception study on the newly recorded stimuli.

**Table 1:** Acoustic properties of the auditory stimuli in the Auditory experiment across mask conditions.

	**Without mask**			**With mask**		
	**Medial Mean (SD)**	**Edge Mean (SD)**	***t*** **-test, *p*-value**	**Medial Mean (SD)**	**Edge Mean (SD)**	***t*** **-test, *p*-value**
Sentence length (ms)	2200.4 (99.9)	2162.7 (206.4)	0.84, *p* = 0.4	2222.3 (134.2)	2167.6 (211.9)	1.1, *p* = 0.3
Word duration (ms)	335.0 (57.9)	555.1 (49.8)	14.1, *p < * 0.001	337.9 (71.2)	556.6 (51.6)	−12.1, *p < * 0.001
Pitch range (Hz)	−22.2 (15.4)	−46.7 (14.3)	5.7, *p < * 0.001	−13.9 (18.8)	−46.6 (14.4)	5.9, *p < * 0.001
Tonal event	—	L%	—	—	L%	—

#### Perception Validation of the Stimuli: Perception Study With Adults

The newly recorded 96 utterances (4 pseudowords x 6 sentences x 2 word positions x 2 mask conditions) were tested in two AX perception tasks: an intelligibility task and a direct-question task. The participants heard a pair of utterances, after which their response was recorded (*via* keyboard). In the intelligibility task, the participants were asked to answer which utterance was the most intelligible. The participants had three response options: the first utterance, the second, or both. In the direct-question task, the participants were asked to identify the utterance produced with a mask. Here, besides the three response options, we added a fourth response: none. We included different utterance pairs (i.e., with a mask—without a mask) and same utterance pairs (i.e., with a mask—with a mask, without a mask—without a mask). Each AX task had a total of 144 trials (96 different trials and 48 same trials), including order counterbalancing and trial repetition (each pair was presented two times). The trials were randomly presented to the participants on a laptop, using the SuperLab v. 6 software (Cedrus Corporation), while wearing Senheiser headphones (Model HD 558) in a quiet room. Since the goal was to obtain naïve participants' responses, the tasks did not include a training phase. We analyzed the percentage of occurrence of each response and the participants' reaction times (in milliseconds).

A total of 37 participants were included in the AX perception tasks: 20 for the intelligibility task (14 females, 6 males; age range = 17–31 years; mean age = 20.45; SD = 4.06) and 17 for the direct question task (10 females, 7 males; age range = 18–30; mean age = 20.88; SD = 3.20). The participants were monolingual speakers of European Portuguese from the region of Lisbon, and all reported normal hearing. In addition to a consent form, the participants filled in a questionnaire, including sociolinguistic information.

**Figure 1:**
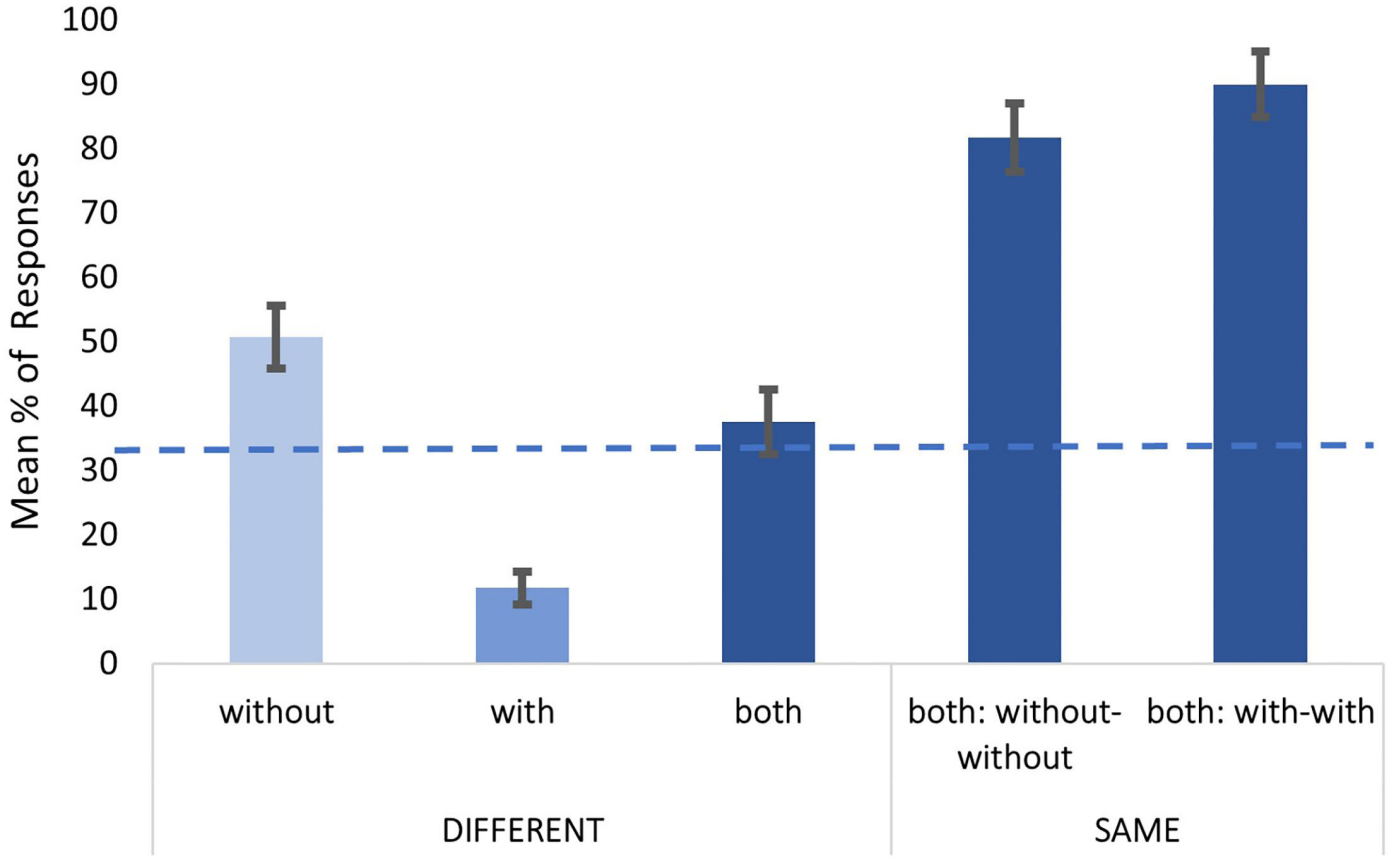
Responses observed in the intelligibility task, for different and same trials. Error bars represent standard error of mean (+/−1). The dashed line represents the chance level (33.33%).

**Figure 2:**
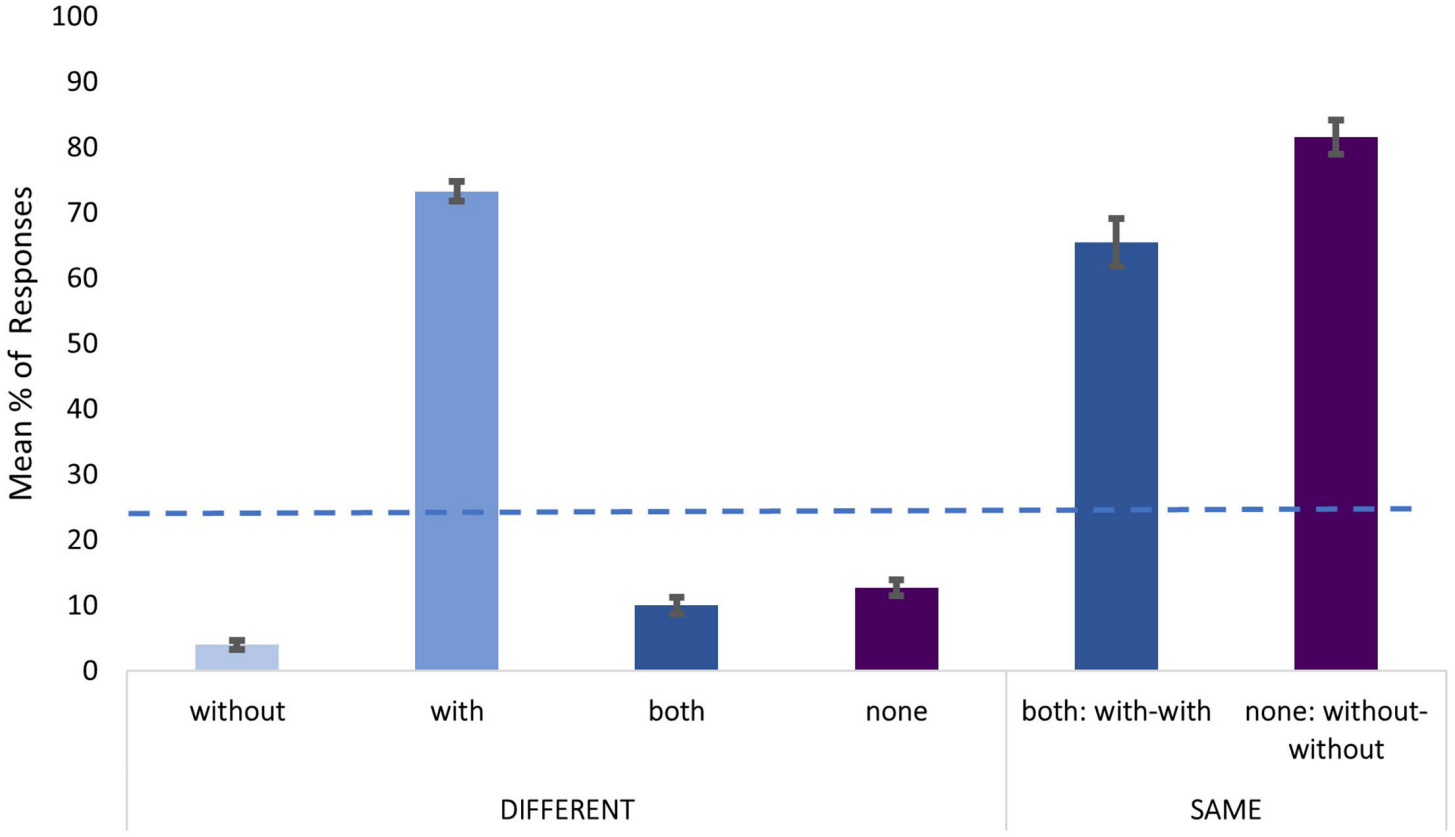
Responses observed in the direct-question task across different and same trial pairs. Error bars represent standard error of mean (+/– 1). The dashed line represents the chance level (25%).

The AX tasks were analyzed separately for different and same pairs. For different pairs, responses were recoded as to whether the participants were choosing the without-mask utterance (*without*), the with-mask utterance (*with*), or both utterances in the pair (*both*). In the direct-question task, there was a further option, *none*. For the same pairs, the same recoding was used. Figures 1, 2, respectively, show the responses obtained in the intelligibility and the direct-question tasks. To compare the distribution of responses with a uniform distribution (i.e., a chance level), a Chi-square goodness-of-fit was conducted. Results for the different pairs in the intelligibility task revealed that the distribution of the participants' responses is significantly different from the chance level [33.33%; χ^2^ = 495.98 (2), *p* < 0.001]. Specifically, the participants were choosing utterances without the mask as more intelligible in 50.7% of the trials (against a chance level of 33.33%). Similarly, for the same trials, the participants were choosing *both* over 80%, suggesting that the participants correctly observed no difference between without-without, and with-with pairs. A Mixed-model analysis in lmerTest package (Kuznetsova et al., 2017) in R (R Core Team, 2020) on reaction time for different pairs is in line with response distribution. The participants were fastest when choosing *without* (M = 2449.1 ms; set as a reference), in comparison to *with* (ß = 103.5, SE = 21.8, *t* = 4.7, *p* < 0.001), and *both* (ß = 131.5, SE = 31.4, *t* = 4.1, *p* < 0.001). Reaction time did not differ between *with* and *both* (*t* = −0.8*, p* = 0.6).

Regarding the direct-question task, results for the different pairs revealed that the distribution of the participants' responses is significantly different from the chance level [25%; χ^2^ = 502.15 (3), *p* < 0.001]. Specifically, the utterances produced with a mask were clearly identified, with 73.2% correct responses in the different pairs. Identification was also successful in the same pairs, with 65.4% correct answers when both sounds were produced with a mask, and 81.6 % correct answers when both sounds were produced without a mask. Again, the participants were faster when correctly identifying *with* as a response (M = 2422.5 ms; set as a reference), in comparison to other incorrect responses (ß = 229.9, SE = 47.5, *t* = 4.8, *p* < 0.001).

In summary, adults listeners perceived acoustic differences between with- and without-mask recordings and found utterances without the mask as more intelligible. Thus, we observed that adults are sensitive to acoustic changes produced by a face mask, which raises the question whether such differences might impact infants' speech processing, in particular their word segmentation ability.

#### Procedure

We followed the same procedure as in the original study (Butler and Frota, 2018), but with the newly recorded stimuli. Before the experimental session, the parents had filled in the consent form. The infants were seating on their caregivers' laps, facing a monitor (Acer), with speakers (Genius) placed behind the monitor, while a camera (Logitech) was placed above the monitor and recorded the experimental session. Stimuli were played at ~70 (+/−5) dB intensity. Each trial began with a baby-friendly image, serving as an attention getter. Once the infant fixated the image for 2 consecutive s, the trial began with a red display paired with a sound file. The trial was infant controlled, i.e., the trial stopped if the infant looked away from the screen for more than 2 s, or if the sound file finished. After the trial ended, the attention getter appeared.

For the familiarization phase, the target words were paired, such that half of the infants were familiarized with FUL—QUEU, and another half with PIS—SAU. Thus, two pseudowords always served as familiar targets in the test phase, while the other two pseudowords were unfamiliar to the infants. The position of the target word within the utterances was also counterbalanced (i.e., for half the infants, FUL was presented in the medial position, and, for the other half, FUL was presented at the edge, and so on). Edge vs. medial presenting order was counterbalanced across the infants. Importantly, edge and medial passages were alternating during the familiarization phase. In the original study, the familiarization stopped after having 25 accumulated s of looking time to each passage (i.e., edge and medial). Here, as the average length of the passages, overall, was slightly longer (~2 s) than in the original study, we decided to proportionally increase the accumulated looking time to 28 s. After the familiarization finished, it was immediately followed by the test phase. The test phase trials were from the original study without any alteration, and they consisted of 15 examples of one target word with a 500-ms pause in between repetitions. There were four of these sound files, two with target words heard during the familiarization phase, and two with target words unfamiliar to the infants. Each trial was presented three times. Presentation was randomized and split into 3 blocks, with four trials each, so that each target word was presented one time before any target word was heard for the second time, and all the target words were presented two times before any target word was heard for a third time. The maximum number of test trials was 12.

The infants' orientation to the screen was online coded by an experimenter, using the LOOK software (Meints and Woodford, 2005). The experimenter was blind to the experimental conditions and wore headphones playing masking music.

### Results

The infants' online-coded looking time was extracted. A subset of data (20%) was offline coded by other experimenter who was also blind to experimental conditions. We observed high inter-coder correlation in coding (Pearson's *r* = 0.96, *p* < 0.001); thus, the online-coded data were used in further analysis. As in Butler and Frota (2018), the segmentation data were analyzed by means of ANOVA.

#### Familiarization Phase

Mean-looking time in the familiarization phase is given in Table 2. The infants' looking time was analyzed in a 2 (familiarization trial: edge and medial)-x-2 (masked condition: without and with) ANOVA to test any potential attentional differences in the familiarization phase. Results revealed no main effects or interaction (Fs <2.4, *ps* > 0.1).

**Table 2:** Mean-looking times for the familiarization phase in the auditory experiment.

**Mask condition**	**Target-position**	**Mean looking time in**
		**seconds (SD)**
With mask	Edge	31.5 (3.1)
	Medial	33.1 (3.6)
Without mask	Edge	31.3 (2.6)
	Medial	32.2 (3.9)

#### Test Phase

**Figure 3:**
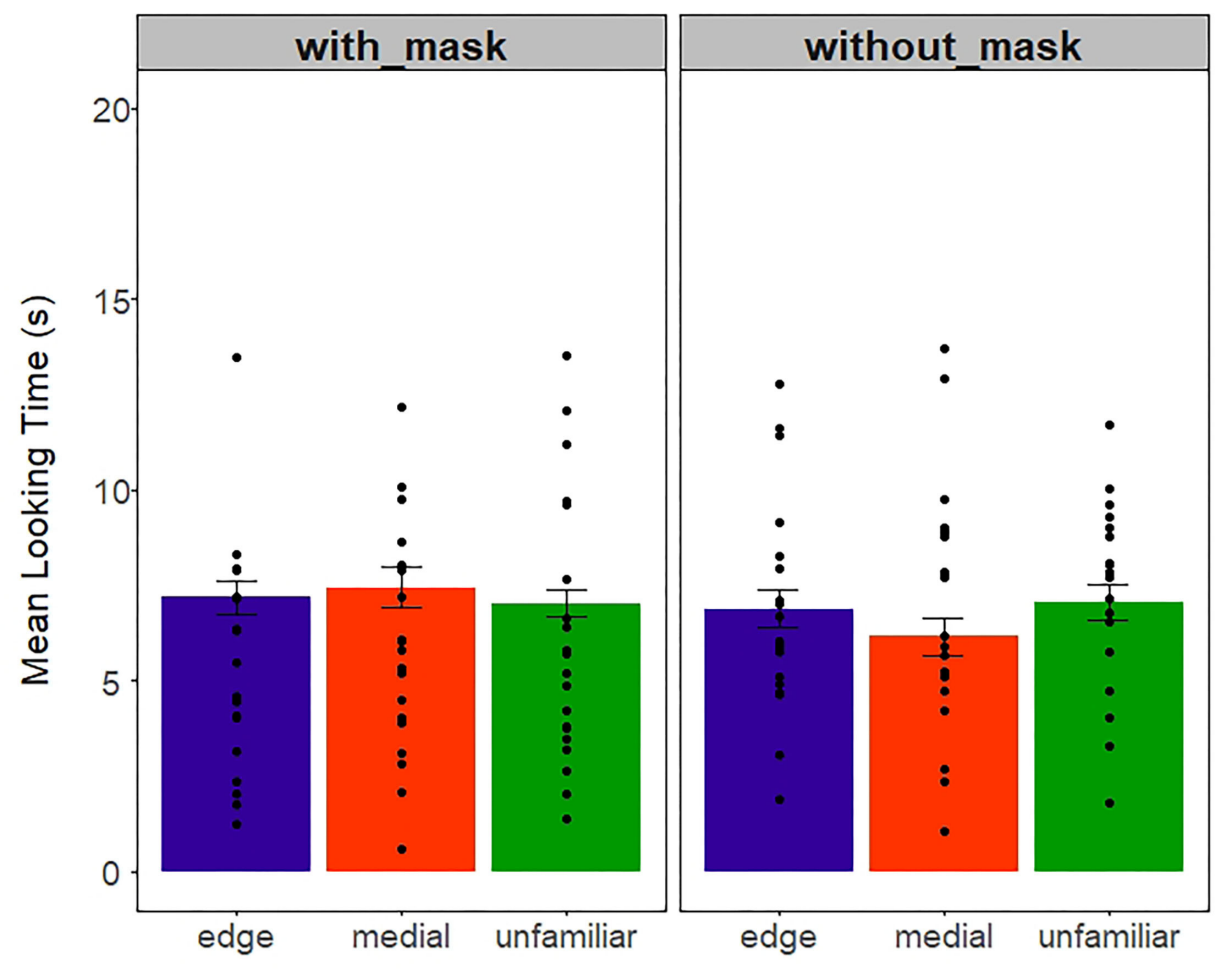
Mean-looking time (s) in the auditory experiment across mask and word-type conditions. Dots represent individual data points. Error bars represent 1+/– standard error of mean.

Almost all the infants provided data for all 12 trials. Only two infants in the without-mask condition did not provide data for the third block. The infants' looking time was averaged across edge, medial, and unfamiliar test trials; thus, each infant contributed with 3 data points. Figure 3 depicts mean looking time to edge, medial, and unfamiliar test trials for the with- and without-mask conditions. A mixed 2 (with and without a mask)-x-3 (edge, medial, unfamiliar) ANOVA revealed no significant main effects nor interactions (all *F*s < 1.03, all *p*s > 0.31).

**Figure 4:**
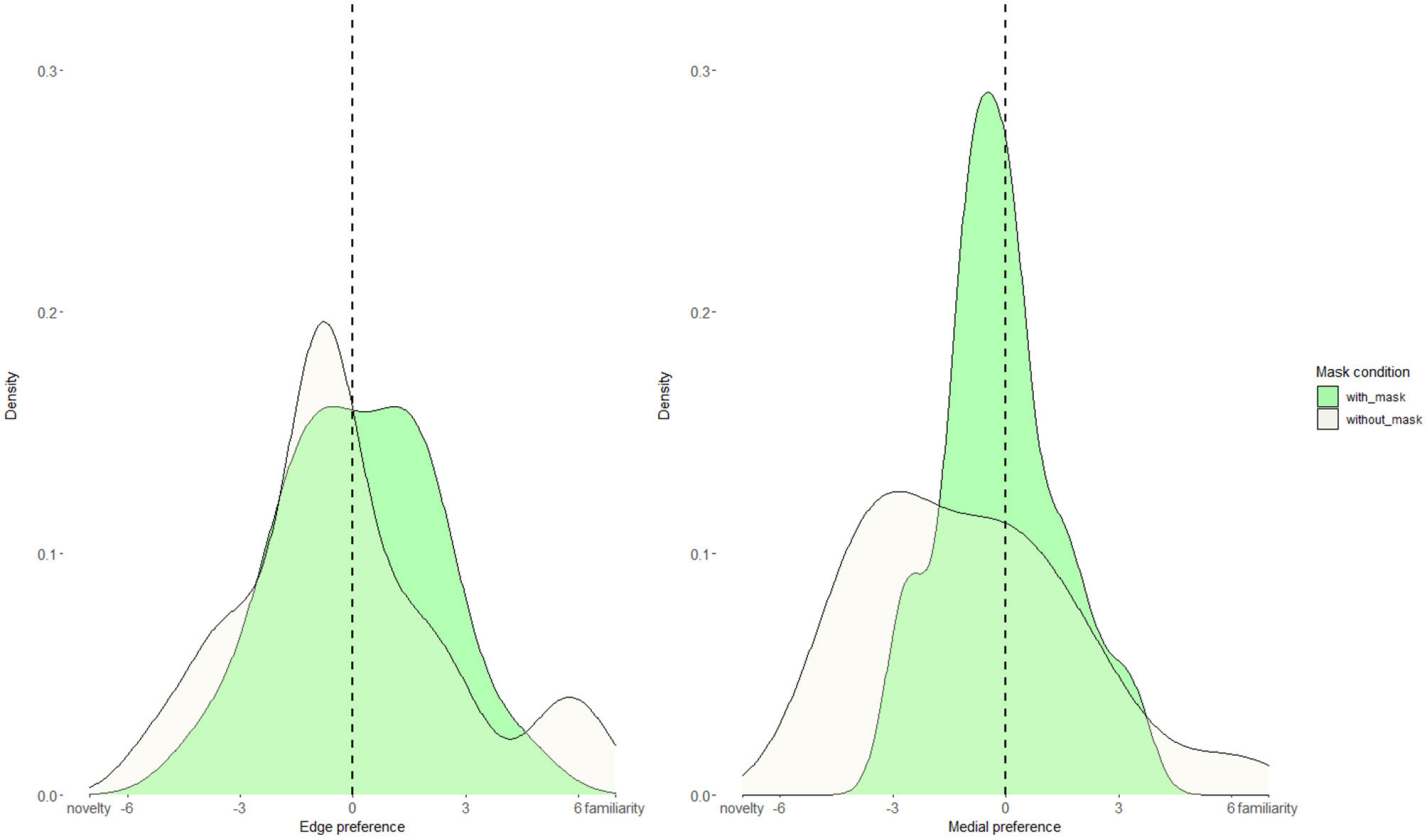
Density distribution plots for edge and medial looking preferences in with-mask (green) and without-mask (beige) conditions. The dashed line represents no preference.

Butler and Frota (2018) observed that infants attended more to edge than to unfamiliar test trials. However, our mean-looking-time analysis did not reveal such a pattern. Given this discrepancy in results, we examined whether most infants do not demonstrate the edge preference, or it might be that some infants do demonstrate it while others demonstrate a preference for unfamiliar, thus providing a null effect. To do so, we calculated the individual looking time difference (i.e., preference) between edge and unfamiliar, and medial and unfamiliar. Specifically, for each infant, we subtracted the mean-looking time to unfamiliar trials from the mean-looking time to edge/medial trials. Each infant provided one data point for edge-unfamiliar preference, and one data point for medial-unfamiliar preference. Thus, if an infant looks longer to edge/medial than to unfamiliar trials, then the preference value is positive, but if he or she looks more to unfamiliar than to edge/medial, then the preference value is negative. If the preference value is around zero, then looking time to edge/medial and unfamiliar is not different. We plotted these individual preferences as density plots (Figure 4) separately for edge and medial using the ggplot package (Wickham, 2016) in R (R Core Team, 2020). Using the geom_density function, we obtained density plots separately for with- and without-mask conditions, with an optimizing bandwidth parameter. Finally, we run a Kolmogorov-Smirnov test that revealed no difference for preference distribution across with- and without-mask conditions (edge *p* = 0.5, medial *p* = 0.1).

The infants' preferences were also analyzed in relation to their concurrent CDI and CSBS scores, as well to the infants' exposure to a mask and number of people they regularly interacted with on a weekly basis. Mixed model analyses were run separately for CDI, CSBS, and exposure to a mask and number of people. We separately modeled the infants' edge and medial preferences, with mask condition as a fixed effect, in addition to the mentioned measures, while the participants were kept as a random effect. None of the measures were significant in relation to the infants' edge preference (all *t*s < 1.1, all *p*s > 0.15). However, for the medial preference, we observed that a higher percentile on CSBS speech scale is related to higher medial preference over unfamiliar (intercept = 0.007, *B* = *0.07, t* = *2.8, p* = *0.008*).

### Discussion

The results of the *Auditory experiment* show that looking times to familiar target words, whether in utterance-edge or utterance-medial position, and unfamiliar target words were not significantly different, regardless of mask condition. Moreover, the distribution of the infants' preferences to familiar and unfamiliar target words also did not differ across with and without a mask. In other words, we found no evidence for word segmentation either at utterance-edge position or at utterance-medial position, as well as no difference in segmentation abilities when speech was produced with a face mask. These findings seem unexpected on two grounds. First, earlier work using the same experiment design had found successful segmentation at utterance-edge position from 4 months of age (Butler and Frota, 2018). It might thus be expected that 7–9-month-old infants would demonstrate word segmentation for words in utterance edges, namely, in the without-mask condition, which is equivalent to the *Auditory* experiment from Butler and Frota (2018). Second, face masks have been shown to degrade the acoustic signal and reduce speech intelligibility (e.g., Rahne et al., 2021; Thibodeau et al., 2021). Alterations in the acoustic signal were patent in our stimuli (Cruz et al., 2022), and were clearly perceived by adult speakers. However, there was no direct and momentary effect of mask use in the infants' word segmentation. To further test the potential impacts of mask use and COVID-related changes on word segmentation abilities, an *Audiovisual experiment* was conducted.

## Audiovisual Experiment

The design of the *Audiovisual* (AV) experiment followed the design of the *Auditory* experiment, except that, in the familiarization phase, the infants saw and heard the speaker.

### Materials and Methods

#### Participants

Forty 7–9-month-old infants took part in the AV experiment, twenty in the with-mask condition and twenty in the without-mask condition. The mean age was 8.1 months for with a mask (range, 7 months, 4 days to 9 months, 7 days; 9 female subjects) and 8.2 months for without a mask (range, 7 months, 6 days to 9 months, 11 days). The groups did not differ in their age [*t* (1, 38) = 0.5, *p* = 0.6]. Seven more infants were tested, but their data were not included in the analysis due to: being bilingual/bidialectal (2), crying or being fussy (4), and not providing data for at least one block (1). For all the tested infants, the parents reported no health-related issues, and no familial risks for language impairment. All the infants were born full-term (> 37 gestational weeks), with an APGAR score at the 5th min of more than 7, and weight above 2,500 g. The infants were recruited in the larger area of Lisbon, and all were acquiring European Portuguese. Twelve infants were attending a daycare. As, in the *Auditory* experiment, we observed no difference between the infants in their everyday exposure to mask (M_withamask_ = 1.7, M_withoutamask_ = 1.6; meaning that answer number 2, that is, 2 to 5 h of daily mask exposure, was predominant), in the number of people they regularly interacted with on a weekly basis (M_withamask_ = 6.6, M_withoutamask_ = 4.6), or in their scores at CSBS and CDI measures (all *p*s > 0.25).

#### Stimuli

**Figure 5:**
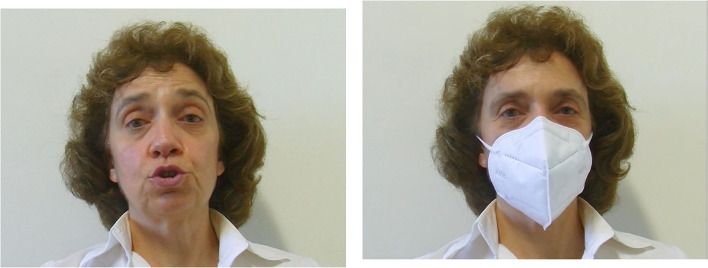
An example frame from audiovisual stimuli for the without- and with-mask conditions.

To create the audiovisual stimuli, we used audio and video recordings captured by the camera. Video recordings were done with a professional JVC camera, model GY-HM11E, in.mov format (a 4:3 aspect ratio, 25 fps). These recordings were occurring simultaneously with the recordings of the auditory stimuli in the *Auditory* experiment. Note that the speaker held the microphone used for the *Auditory* experiment just below the chin-line, and the microphone was not visible in the video recordings. The speaker was recorded sentence by sentence in front of a white background, looking directly to the camera. The speaker was instructed to act/move in an infant-friendly manner. No instructions were given to act exactly the same with and without the mask, since we wanted the speaker to have natural speech manners with and without the mask. Before and after each sentence, the speaker set into a neutral-friendly face expression, closing the mouth. An example of a frame with and without the mask is depicted in Figure 5.

Recorded utterances were edited in Adobe Premier Pro 2021 software to create audiovisual passages. Unlike in the *Auditory* experiment, the visual signal precedes the auditory signal, making the audiovisual utterance longer than the auditory signal. We set as the beginning of an audiovisual utterance at the first movement of an articulatory element (e.g., mouth), or a gesture (e.g., head movement), whereas the end was set when the speaker was back to the neutral face with a closed mouth. To create a pause between utterances, as in the auditory passages, we introduced a still frame that was presented for 500 ms. The still frame was edited in Adobe Premier Pro software using an effect that blended the still frame with the first frame of an utterance. The average duration of audiovisual passages was 22 s. Examples of audiovisual stimuli are available at https://labfon.letras.ulisboa.pt/babylab/early_word_segmentation_behind_the_mask/supporting_materials.html.

A detailed analysis of visual cues in the stimuli, namely, head and eyebrow movements, is given in Cruz et al.'s (2022). Head vertical movements were found to be larger in face-masked speech, whereas eyebrow movement was reduced. Head movements were generally larger at utterance-edge position. In the absence of a mask only, it was found that the location of the largest head movement in the utterance aligned with the word target at the utterance edge. No such alignment was found at utterance-medial position.

#### Procedure

The procedure was similar to the *Auditory* experiment; however, stimuli in the familiarization phase were audiovisually presented. The infants' looking data were collected using the EyeLink 1000 Plus eye tracker. The stimuli were presented on an ASUS monitor, with the same size (22 inches) as the monitor in the *Auditory* experiment. The same loudspeakers as in the *Auditory* experiment were used behind the eye tracker. Trial presentation was done in Experiment Builder software (SR research). The stimuli were played at ~70 (+/−5) dB intensity. The infants were sitting on their caregivers' laps and faced the eye tracker. Before the experiment started, the infants' eye gaze was calibrated and validated using a 5-point calibration system. As before, each trial began with a baby-friendly image, serving as an attention getter. Once the infant fixated the image for 2 consecutive s, a trial began with a red and black checkerboard display paired with a sound file^2^. Given that audiovisual passages were longer than the auditory-only passages (~22 s), we proportionally adapted the accumulated looking time to be 43 s. Everything else was set as in the *Auditory* experiment.

### Results

#### Familiarization Phase

Mean-looking time in the familiarization phase is given in Table 3. First, we analyzed whether the infants exhibited overall attentional differences during the familiarization phase across mask conditions (with and without) and target-word positions (edge and medial).We run the same ANOVA as in the *Auditory* experiment and observed no main effects or interaction (F*s* < 2.7, *ps* > *0.1*).

**Table 3:** Mean-looking times for the familiarization phase in the audiovisual experiment.

**Mask condition**	**Target-position**	**Mean looking time in**
	**video passages**	**seconds (SD)**
With mask	Edge	49.2 (3.6)
	Medial	49.9 (4.5)
Without mask	Edge	48.8 (3.2)
	Medial	47.6 (2.8)

**Figure 6:**
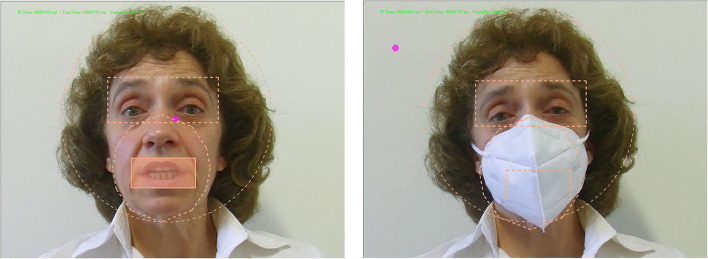
Example frames demonstrating areas of interest.

Apart from overall looking time, we also analyzed the infants' looking patterns during the familiarization phase. Specifically, we created four dynamic areas of interest (AOI) for each video: the head, the mouth, the eyes, the mask (see Figure 6). Areas in pixels were the same across videos (head: 309,255; eyes: 44,415; mouth 16,808; mask 69,254).

**Figure 7:**
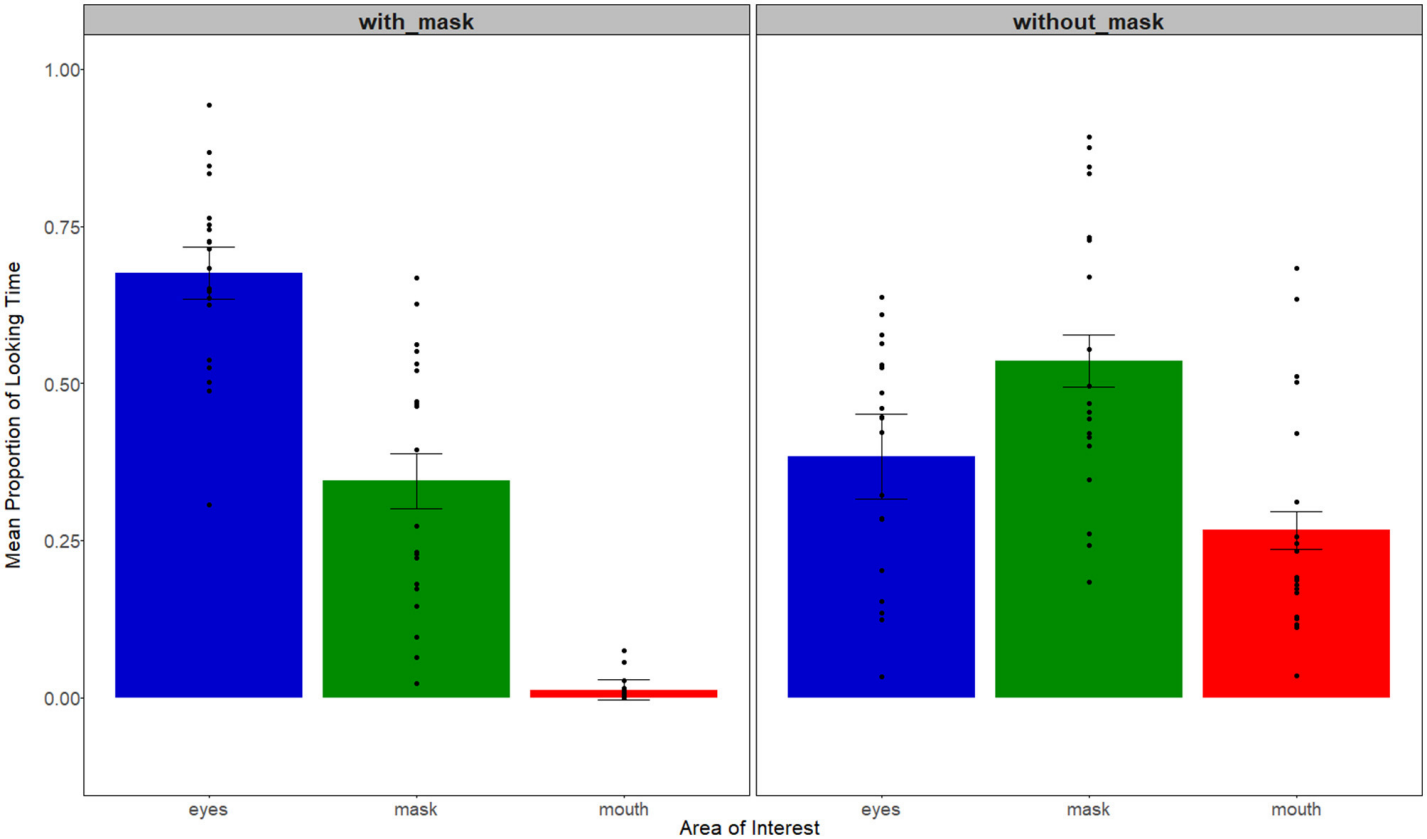
The infants' mean proportion of looking time to the eyes, mask, and mouth across the two mask conditions during the familiarization phase. Dots represent individual data; error bars +/– 1 standard error of mean.

Note that we created the mask area in videos without a mask, and the mouth in the videos with a mask, although, in fact, the infants could not see them. We did so to compare areas across videos with and without a mask, and to see whether the infants' looking patterns changed in presence/absence of a mask. Next, similar to previous studies on infants' looking patterns to the speaking face (e.g., Lewkowicz and Hansen-Tift, 2012; Pons et al., 2015; Pejovic, 2019), we calculated the proportion of looking time to the eyes, the mouth, and the mask in relation to total looking time to the head. Figure 7 depicts the infants' looking patterns during the familiarization phase. A 2 (with and without a mask)-x-3 (eyes, mouth, mask) mixed ANOVA revealed the main effect of AOI [*F*_(2, 114)_ = 56, *p* < 0.001, η^2^ = 0.50], and, more interestingly, a *Mask condition x AOI* interaction [*F*_(1, 114)_ = 29.8, *p*< *0.0*01, η^2^ = 0.34]. To understand the observed interaction, we run a pairwise analysis (Bonferroni-controlled) and found that the infants in the with-mask condition attended more to the eyes than other AOIs (*p*s < 0.001). This pattern was different in the without-mask condition, where the infants attended more to the mask area than the mouth (*p* < 0.001), and the eyes (*p* = 0.01), but with no difference between the eyes and the mouth (*p* = 0.0*8*). Thus, we observed that the infants' looking patterns were affected by face mask use. Interestingly, in the with-mask videos, we observed increased looking to the eyes—an area that was not altered directly by mask usage, suggesting that the infants did not look at the occluded mouth, or the mask, but, instead, they increased their looks to the only available cues, which are provided by the eyes.

#### Test Phase

**Figure 8:**
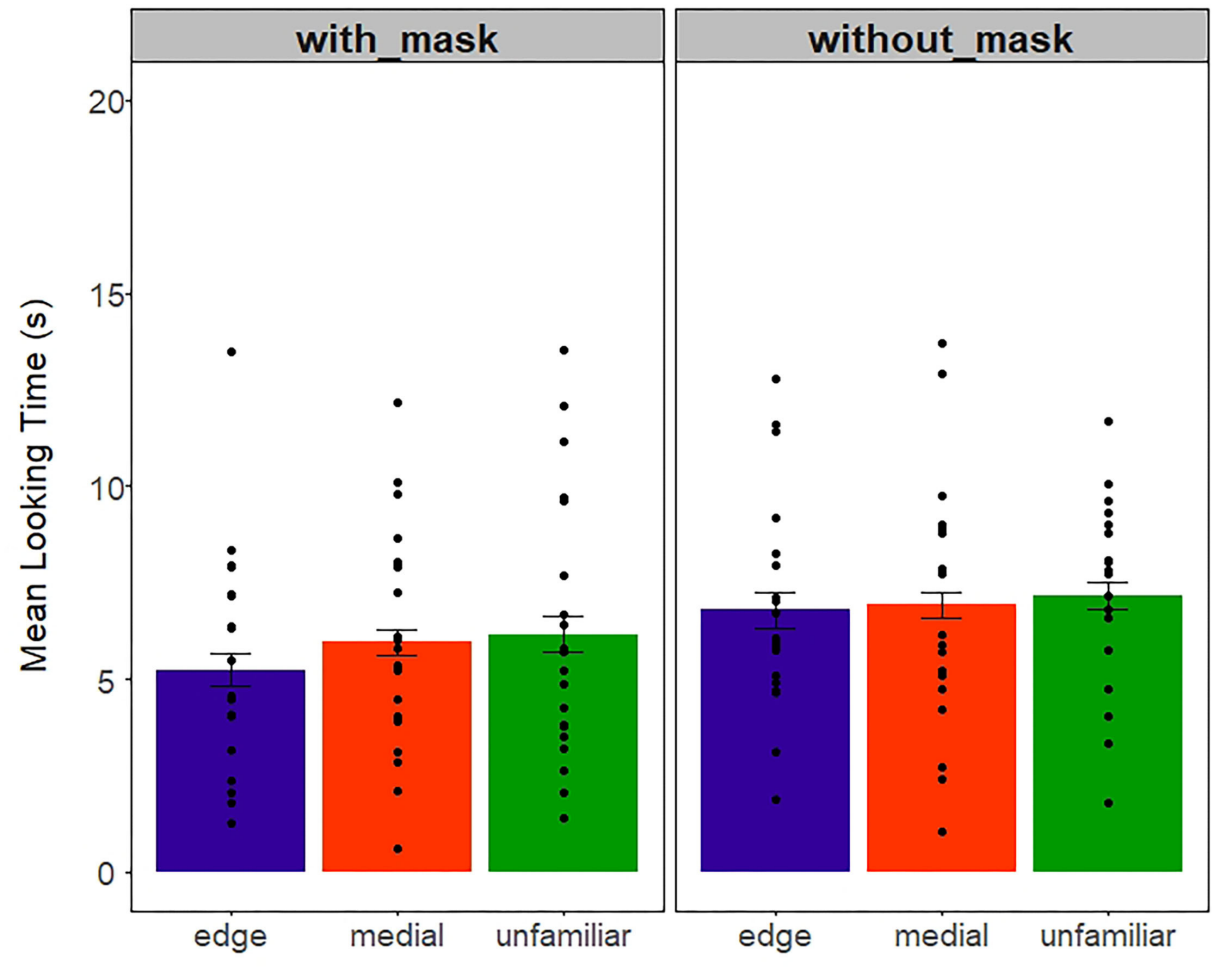
Mean-looking time (s) in the Audiovisual experiment across mask and word-type conditions. Dots represent individual data points. Error bars represent 1+/– standard error of mean.

As in the *Auditory* experiment, we calculated the mean-looking time for each infant across the three word-form conditions (edge, medial, and unfamiliar). Then, the infants' looking time was averaged across edge, medial, and unfamiliar test trials (Figure 8). We conducted a 2 (with and without mask)-x-3 (edge, medial, unfamiliar)-mixed ANOVA that revealed the main effect of mask condition [*F*_(1, 114)_ = 4.63, *p* = *0.0*3, η^2^ = 0.04]. Specifically, the infants attended more to the screen during the test trials when previously familiarized with videos without the mask (M = 6.9), in comparison to being familiarized with videos with the mask (M = 5.7). No other effect reached significance [*F*s < 0.4, *p*s > 0.6].

**Figure 9:**
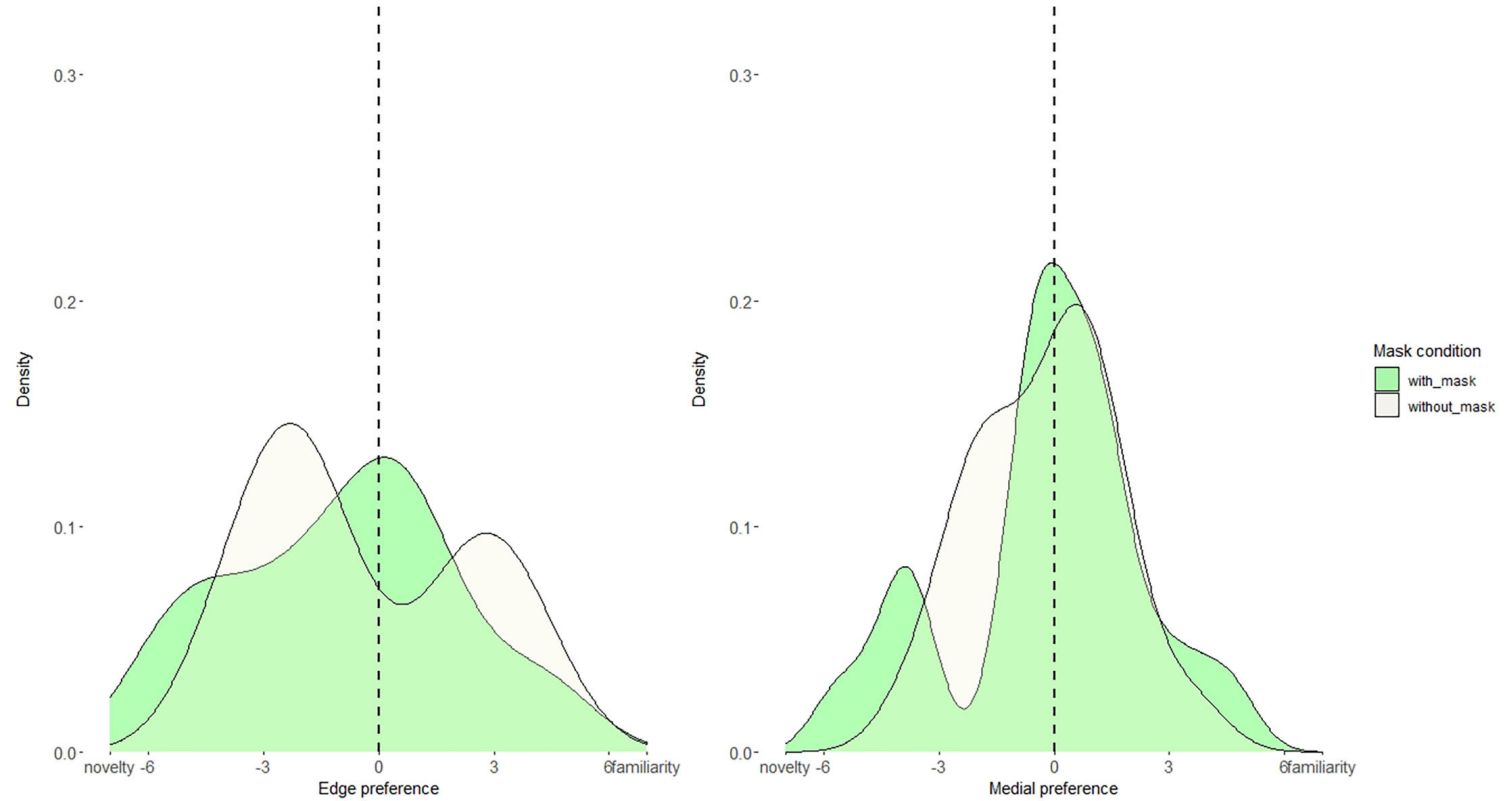
Density distribution plots for edge and medial looking preferences in with-mask (green) and without-mask (beige) conditions.

We again examined the infants' preferences (i.e., the looking time difference) between edge and unfamiliar, and medial and unfamiliar. The distribution of edge-unfamiliar and medial-unfamiliar preferences is plotted in Figure 9, using the method described in Section Results. A Kolmogorov–Smirnov test revealed no differences for preference distribution across with- and without-mask conditions (edge *p* = 0.4, medial *p* = 0.5).

It is important to note that, in the edge preference, we see a bimodal distribution for the without-mask condition, suggesting that infants tend to deploy opposite segmentation strategies (familiarity vs. novelty), which we do not observe in the with-mask condition, or at utterance-medial position. To further investigate a possible difference between the development of segmentation abilities at edge and medial positions, we asked whether there was a difference in preference *magnitude* across conditions, regardless of whether the infants looked more at the familiar word (edge/medial) or the unfamiliar word. We thus transformed the preference values (i.e., edge and medial preferences) into absolute values. An ANOVA on absolute preference as a dependent variable, with the mask condition as a between-subject factor, and position type as a within-subject factor, revealed a significant main effect of position [*F*_(1, 76)_ = 6.7, *p* =0.01, η^2^ = 0.08]. The absolute difference for edge (M = 2.4) was greater than for medial (M = 1.6), indicating that segmentation abilities seem to be developing better for words at edge-utterance position. No other effects were found.

The infants' preference was also analyzed in relation to the infants' concurrent CDI and CSBS scores, as well with the infants' daily exposure to a mask and number of people they regularly interacted with on a weekly basis. Several mixed model analyses were run as in the auditory experiment. None of the measures were significant in relation to the infants' edge or medial preference (all *ts* < 1.2, all *p*s > 0.12).

#### Relation Between Looking Patterns in the Familiarization Phase and Segmentation Abilities

Previous work suggested that infants attending more to visual cues also show better segmentation abilities (Tan and Burnham, 2019), and that adults' segmentation ability increases with increased attention to the eyes (Lusk and Mitchel, 2016). We thus tested whether the infants' segmentation ability was modulated by the visual cues they were attending during the familiarization phase. We ran a series of correlational analyses relating the infants' preference for edge/medial with the infants' proportion of looking time to the eyes/mouth/mask. We observed no significant relation between the infants' preferences and looking patterns in the familiarization phase (all *p*s > 0.2).

### Comparison Between the Audiovisual and Auditory Experiments

To directly compare performance between the two experiments, we ran a mixed ANOVA on the infants' mean-looking time with the between factors *Experiment* (auditory and audiovisual) and *Mask condition* (with and without a mask) and the within-factor word type (edge, medial, unfamiliar). The analysis revealed an interaction between *Experiment x Mask* condition [*F*_(1, 210)_ = 6.3, *p* = 0.01, η^2^ = 0.03]. Further *post hoc* comparisons (Bonferroni corrected) demonstrated that overall infants' looking time was decreased in the *Audiovisual* with mask condition in comparison to *Audiovisual* without a mask (*p* = 0.04) and to *Auditory* with mask condition (*p* = 0.03). No other effects were found.

### Discussion

Like in the *Auditory* experiment, the results of the *Audiovisual* experiment show no evidence for word segmentation either at utterance-edge position or at utterance-medial position, and no difference in segmentation abilities when speech was produced with a face mask. Thus, the *Audiovisual* experiment confirmed the basic findings of the *Auditory* experiment. Other results from the *Audiovisual* experiment, however, differed from the *Auditory* experiment. In *Audiovisual* only, the infants displayed longer looking times for test trials when previously familiarized with speech without the mask than with masked speech. This suggests that audiovisual face-masked speech negatively impacted the infants' general attention during the test phase of the segmentation task. Furthermore, although no evidence for segmentation emerged in the *Audiovisual* experiment, the infants' preferences between edge and familiar showed a bimodal distribution in the without-mask condition, indicating that infants are processing the utterance-edge differently from the utterance-medial position only when speech is audiovisually presented without a mask. In addition, the magnitude of the absolute difference in looking time between edge and unfamiliar was larger than between medial and unfamiliar. In line with previous word segmentation studies (Johnson et al., 2014; Butler and Frota, 2018; Frota et al., 2020), these findings suggest an advantage of the edge position over the medial position, which is consistent with the emergence of word segmentation at the utterance edge first, and was only found in the *Audiovisual* experiment. This set of findings, specific to the *Audiovisual* experiment, adds to previous suggestions that audiovisual information might provide support to infant speech segmentation (de la Cruz-Pavía et al., 2019; Tan and Burnham, 2019).

## Early Word Segmentation And Later Vocabulary Outcomes

In previous work, word segmentation has been suggested to support language learning and predict later language abilities, namely, vocabulary development (Newman et al., 2006; Singh et al., 2012). Although no evidence for word segmentation was found in the current study, we asked whether the infants' segmentation abilities at 7–9 months of age (mean age, 8.2 months) might relate to their later vocabulary development. The infants that performed the word segmentation experiments are part of an ongoing longitudinal study, aiming to track their language and communicative development until 18 months of age. At the moment, later CDI data from 34 infants are available. We thus examined the relation between the infants' performance in the segmentation experiments and their expressive vocabulary at 10–13 months of age (mean age, 12.3 months, 20 female subjects). The results showed a trend whereby the infants that looked more to target words at utterance-edge position than to unfamiliar words [*r* (32) = 0.33, *p* = 0.058] and that looked more at target words at utterance-medial position than to unfamiliar words [*r* (32) = 0.33, *p* = 0.052] have larger later expressive vocabulary outcomes measured with the CDI percentile. This finding is in line with previous studies by indicating that emerging segmentation abilities are signaled by more looks to familiar target words at edge position and medial position than to unfamiliar words (Butler and Frota, 2018; Frota et al., 2020) and by showing a positive relation between better performance at the segmentation task and later vocabulary development.

## Comparison With Segmentation Data and Measures in Pre-Pandemic Time

Considering that, in the current study, we observed no evidence for word segmentation abilities being already well-developed by 7–9 months of age, regardless of word position and auditory or audiovisual speech presentation, we decided to compare the current data from the *Auditory* experiment without a mask with the earlier segmentation data collected in 2016–2017 in the same lab by the same team, using the same procedure (Butler and Frota, 2018). Note that the acoustic properties of the familiarization stimuli of the current study exhibit the same distinguishing features between edge and medial positions as in the original study (as shown in Section Stimuli), and the same test trials from the original study were employed. From the original study, we selected the infants between 7 and 9 months of age (*N* = 19) and compared their data with the *Auditory* experiment without the mask data (*N* = 19). Given that we observed an age difference between studies (the infants in the new study are younger than in the old study, respectively, M = 8.2, M = 8.7, *p* = 0.03), we conducted a regression analysis on the infants' mean-looking time with the predictors *Study* (original and current), word type (edge, medial, unfamiliar), and age as a covariate. The model was significant [*R*^2^ = 0.15, *F*_(6, 107)_ = 3.2, *p* = 0.006]. We observed an interaction between study and word type [*F*_(2, 107)_ = 3.21, *p* =*0.0*3]. Further comparisons indicated that the looking time for edge was significantly higher than for unfamiliar only in the original study (*p* = 0.0002), but not in the current study (*p* = 0.9).

Since we did not have information on CDI and CSBS measures from the original study, we decided to compare the development of the infants from the current study with the normative data for those questionnaires (Frota et al., 2016; Filipe et al.^3^). Thus, we were able to directly compare the development of the infants born in pandemic times with the infants' development occurring in pre-pandemic time. For CDI, we have compared concurrent CDI for 8 and 9 months of age (N_8_ = 46, N_9_ = 18)^4^ with the normative CDI data (N_8_ = 21, N_9_ = 40), controlling for gender. A series of non-parametric Wilcoxon tests were run, and we observed no differences in CDI measures on comprehension or a production score (all *p*s > 0.1).

Regarding CSBS measures, we compared concurrent scores for 7, 8, and 9 months of age from the current data set (N_7_ = 23, N_8_ = 42, N_9_ = 9), with norming data (N_7_ = 21, N_8_ = 17, N_9_ = 14). In a mixed-models analysis, we separately modeled Social, Speech, Symbolic, and Total scores with age, gender, and dataset as fixed factors, while the participants were set as a random factor. We observed that the infants from the current study were, overall, lower (M = 11.3) than the norming data (M = 13.1; t = −2.6, *p* =0.01) on the Social scale. Similarly, we observed lower total scores in the current study (M = 20.8) than in the norming study (M = 23.7; *t* = −2.4, *p* = 0.01).

## General Discussion

The current study examined the impact of mask use and COVID-related changes in early word segmentation abilities. The study had three main goals: 1) examine the momentary impact of mask use in early word segmentation abilities; 2) establish whether audiovisual cues might provide additional support to facilitate the infants' word segmentation; and 3) explore whether COVID-19 related changes in communication and interaction, which include continued exposure to altered speech cues, might have impacted the development of word segmentation. The first and second goals were addressed by conducting two word-segmentation experiments: *auditory* and *audiovisual*, within which we manipulated the presence or absence of a face mask. Both experiments followed a prior study done on European Portuguese infants (Butler and Frota, 2018). We observed no evidence for word segmentation in 7–9-month-old infants in the current study, unlike in the original study, where successful segmentation was found at utterance-edge position from 4 months of age. In addition, a study on the relation between the infants' segmentation abilities at 7–9 months of age, shown in the word segmentation experiments, and their later expressive vocabulary outcomes was conducted. We observed a positive relation between better performance at the segmentation task and later vocabulary development. The third goal was addressed by means of a direct comparison between the current infant data and equivalent measures obtained in pre-pandemic time. Besides confirming that the infants from the current study did not display developed segmentation abilities at the utterance edge, unlike the infants from the pre-pandemic study, results also showed that the infants from the current study had lower scores on measures of communicative development. In the next paragraphs, we discuss in more detail the present findings.

Word segmentation is an important milestone in language acquisition. Recent studies have indicated that speech segmentation in adults and infants is supported by visual speech cues (Mitchel and Weiss, 2014; Lusk and Mitchel, 2016; Tan and Burnham, 2019). We investigated whether word segmentation could be altered if visual speech cues are occluded by a face mask. In addition, the acoustic signal is degraded in face-masked speech (e.g., Rahne et al., 2021; Thibodeau et al., 2021; Cruz et al., 2022), and no study so far has addressed whether word segmentation might be affected by a degraded acoustic signal. Beyond the direct experimental manipulation contrasting speech produced with and without a mask, it might also be the case that continued exposure to altered speech cues, together with other COVID-related changes, might have affected the development of word segmentation abilities.

Alterations in the acoustic signal were patent in our stimuli, and adults were sensitive to them. Specifically, they considered speech produced without the mask as more intelligible than face-masked speech, and correctly identified speech that is produced with a mask. In addition to the occlusion of the mouth area, mask use impacted other visual speech cues, such as head and eyebrow movements. In particular, both acoustic and visual cues were found to be more prominent in utterance-edge position than in utterance-medial position, and the contrast between edge and medial was stronger without a mask than with a mask. Therefore, the absence of successful word segmentation either in the *auditory* or the *audiovisual* experiment, and the absence of a difference between the without-a-mask and with-a-mask experimental conditions, cannot be ascribed to the lack of contrasting cues in the speech signal. Moreover, the absence of a difference between the without-a-mask and with-a-mask experimental conditions suggests that speech delivered with a face mask had no direct and momentary effect on infant word segmentation.

Given that no evidence for segmentation was found both in the auditory and audiovisual experiments, the current findings suggest that audiovisual cues were not enough to provide additional support to infants' word segmentation. However, several findings from the *audiovisual* experiment indicate that the audiovisual impact of face-masked speech was stronger than the auditory impact. The infants were less attentive to language when it was visually delivered through a mask. Furthermore, unlike in the *auditory* experiment, the absence of a mask in the familiarization phase divided the infants in their strategy to segment words at the utterance edge. Almost half of the infants developed edge, and, the other half, unfamiliar preference, showing that they were processing the utterance edge differently from the utterance-medial position only when speech was audiovisually presented without a mask. This was further supported by the larger difference in looking time between edge and unfamiliar than between edge and medial. These findings are in line with two suggestions from previous studies. On the one hand, they confirm the advantage of utterance-edge position, a prosodically prominent position, over utterance-medial position in the development of early word segmentation abilities (Johnson et al., 2014; Butler and Frota, 2018; Frota et al., 2020). On the other hand, they support the idea that audiovisual speech cues might facilitate infant speech segmentation (de la Cruz-Pavía et al., 2019; Tan and Burnham, 2019).

Yet other patterns in our findings speak to the role of audiovisual cues. We observed that, depending on mask condition, the infants' looking patterns changed. In response to the face mask, the infants dominantly spent more time looking at the eyes, whereas, without the mask, the infants alternated between the eyes and the mouth (in line with Cruz et al., 2020; Pejovic et al., 2021; Sekiyama et al., 2021). This suggests that, when articulatory cues are occluded, infants do not attend to the occluded area but redirect their attention to the available visual cues, namely, those provided by the eyes. Interestingly, we found that the infants' segmentation abilities were not related to their looking patterns. A similar finding was observed in a much smaller sample sized study, in 7.5-month-old infants (Tan and Burnham, 2019). However, adults' segmentation abilities are related to increased attention to the eyes (Lusk and Mitchel, 2016). The difference in adult and infant data suggests that, even when infants attend to visual segmentation cues (like the eyes in the masked condition), they might not fully grasp it as a cue for segmentation. This explanation is limited to infants up to 7–9 months of age, and future research should address when and how in development infants are able to take full advantage of visual cues for word segmentation.

The relation between the infants' performance in the segmentation experiments and their later expressive vocabulary outcomes was also inspected. Interestingly, those infants that are already showing the excepted segmentation pattern, with more looks to familiar target words than to unfamiliar target words (Butler and Frota, 2018; Frota et al., 2020), are the ones exhibiting larger expressive vocabularies in line with previous work, showing that word segmentation abilities support vocabulary development (Newman et al., 2006; Singh et al., 2012). Thus, although no evidence for word segmentation was found in the current study, the patterns of emerging segmentation abilities and their relation to later language outcomes suggest that word segmentation abilities, albeit delayed in the infants from the current study, are developing, following a similar path to that found in earlier studies.

One might be puzzled by the unexpected finding that we observed no evidence for word segmentation at 7–9 months of age in the current study, whereas, in Butler and Frota (2018), the infants demonstrated a segmentation ability for words at the utterance edge from 4 months of age. A direct comparison between the pre-pandemic data from Butler and Frota (2018) and the current *Auditory* experiment data (without-mask condition) confirmed that the infants in the original study segmented words at the utterance edge, but not infants from the current study. In another study, with data collected before the pandemic (Frota et al., 2020), word segmentation skills in 6–26-month-old infants that are at risk for language impairment were examined, employing the same stimuli and paradigm as in Butler and Frota (2018). It was observed that the infants at risk for language development displayed a segmentation ability for words at the utterance edge. Therefore, it might be the case that language/communication development might have been affected by COVID-19 changes, which included continued exposure to altered speech due to mask use together with changes in everyday activities related to social interactions. In Portugal, and specifically in the Lisbon area, there were several long periods of lockdown during 2020 and 2021, and other extended periods with severe restrictions on social interactions. Mask use has been obligatory in public places since May 2020 (and in outside areas between October 2020 and October 2021). Although no direct relation was found in our experimental data between the infants' segmentation abilities and their degrees of daily exposure to a face mask, or the number of people they regularly interacted with, the infants from the current study scored lower on measures of communicative development in comparison with normative data for the same measures (which were collected before COVID-19). Indeed, we observed a lower score on the social scale and a lower total score on the CSBS in the current dataset. The scores did not differ for the CDI measures, but it is important to note that variability in the CDI data in this age is much lower than in the CSBS, which might explain why we did not observe differences on the CDI measures. Our findings thus suggest that the infants might have been exposed to less social interaction than before, with impact on their communicative development. Notably, less than one third of the infants in the current study were attending daycare. A recent study has observed that children born and raised during COVID-19 have a lower score on overall cognitive development than the infants' born and raised before the pandemic (Deoni et al., 2021). Another study suggested that attending nursery in contrast to staying at home during the pandemic correlated with better language outcomes (Davies et al., 2021). However, even though we observed lower scores on communicative development in the current data, we did not observe a clear relation between the CSBS and segmentation abilities. Moreover, we do not have information on social interactions for the norming study, and it remains open whether social contact was, indeed, significantly decreased for the infants in the current study. Future studies are crucial to address how cognitive, social, and language development was affected during the pandemic, and how they interact.

In summary, the present findings suggest an overall effect of the pandemic on early segmentation abilities, which is probably multifactorial, extending beyond the pervasive use of face masks to changes in communication and social interactions. The lack of evidence for successful word segmentation, especially at the utterance-edge position, indicates that segmentation abilities are delayed in the infants born during the COVID-19 pandemic. Importantly, we observed that audiovisual speech presentation, without a mask, led to changes in the infants' segmentation ability, but not sufficient to support successful word segmentation. The advantage for segmentation of words at the utterance-edge position in audiovisual speech, together with a positive relation between better performance at the segmentation task and later vocabulary development, suggests that segmentation abilities in the 7–9-month-old infants in the present study, albeit delayed, are developing, following a similar path to that found in earlier pre-pandemic studies. Further research is needed, in particular longitudinal studies, to determine how language development for the infants born during the COVID-19 pandemic proceeds, what areas might be affected, and what strategies might be used to possibly compensate for the reduced exposure to social interaction and multimodal language cues.

## Data Availability Statement

The raw data supporting the conclusions of this article will be made available by the authors, without undue reservation.

## Ethics Statement

The studies involving human participants were reviewed and approved by School of Arts and Humanities Research Ethics Board. Written informed consent to participate in this study was provided by the participants' legal guardian/next of kin. Written informed consent was obtained from the individual(s) for the publication of any potentially identifiable images or data included in this article.

## Author Contributions

SF conceived and designed the study with the help of MV. SF, JP, CS, and MV worked on the stimuli. JP, MC, and CS implemented the experiments and collected the data. JP and CS coded the looking data. MC and MV analyzed the acoustic stimuli. MV did the prosodic analysis. MC analyzed the audiovisual stimuli. JP and MC performed the statistical analyzes. SF supervised the study. SF and JP wrote the manuscript. All authors contributed to the manuscript revision, read and approved the submitted version.

## Funding

This research was developed within the UIDB/00214/2020 project, funded by Fundação para a Ciência e a Tecnologia (FCT), Portugal and the PTDC/LLT-LIN/29338/2017 project (PI SF) funded by FCT in conjunction with the European Regional Development Fund from the EU, Portugal 2020 and Lisboa 2020.

## Conflict of Interest

The authors declare that the research was conducted in the absence of any commercial or financial relationships that could be construed as a potential conflict of interest.

## Publisher's Note

All claims expressed in this article are solely those of the authors and do not necessarily represent those of their affiliated organizations, or those of the publisher, the editors and the reviewers. Any product that may be evaluated in this article, or claim that may be made by its manufacturer, is not guaranteed or endorsed by the publisher.

## Footnotes

^1^Note that CSBS questionnaires were incomplete for two subjects, one from each condition.

^2^Note that we piloted the same red display as in the auditory experiment, but the infants, probably because they were familiar with a talking face, lost interest to attend to a far-less-attractive display. Because of this, we changed the red display to a black and red checkerboard.

^3^Filipe, M., Severino, C., Vigário, M., and Frota, S. (In preparation). *Adaptation and Validation of the European Portuguese Communication and Symbolic Behavior Scales Infant-Toddler Checklist*.

^4^Note that, here, we included only concurrent CDI measures; thus, those infants that were tested at 7 months of age were not included because their CDI could not be assessed (the CDI age range starts at 8 months of age).
